# Botulinum Toxin Type A for the Treatment of Neuropathic Pain in Neuro-Rehabilitation

**DOI:** 10.3390/toxins7072454

**Published:** 2015-06-30

**Authors:** Domenico Intiso, Mario Basciani, Andrea Santamato, Marta Intiso, Filomena Di Rienzo

**Affiliations:** 1 Unit of Neuro-rehabilitation, Istituto di Ricovero e Cura a Carattere Scientifico (IRCCS) Casa Sollievo della Sofferenza Hospital, San Giovanni Rotondo (FG) 71013, Italy; E-Mails: m.basciani@operapadrepio.it (M.B.); marta.intiso@gmail.com (M.I.); f.dirienzo@operapadrepio.it (F.D.R.); 2 Department of Physical Medicine and Rehabilitation, OORR Hospital, University of Foggia, Foggia 71121, Italy; E-Mail: andrea.santamato@unifg.it

**Keywords:** pain, neuropathic pain, botulinum toxin, rehabilitation

## Abstract

Pain is a natural protective mechanism and has a warning function signaling imminent or actual tissue damage. Neuropathic pain (NP) results from a dysfunction and derangement in the transmission and signal processing along the nervous system and it is a recognized disease in itself. The prevalence of NP is estimated to be between 6.9% and 10% in the general population. This condition can complicate the recovery from stroke, multiple sclerosis, spinal cord lesions, and several neuropathies promoting persistent disability and poor quality of life. Subjects suffering from NP describe it as burning, itching, lancing, and numbness, but hyperalgesia and allodynia represent the most bothersome symptoms. The management of NP is a clinical challenge and several non-pharmacological and pharmacological interventions have been proposed with variable benefits. Botulinum toxin (BTX) as an adjunct to other interventions can be a useful therapeutic tool for the treatment of disabled people. Although BTX-A is predominantly used to reduce spasticity in a neuro-rehabilitation setting, it has been used in several painful conditions including disorders characterized by NP. The underlying pharmacological mechanisms that operate in reducing pain are still unclear and include blocking nociceptor transduction, the reduction of neurogenic inflammation by inhibiting neural substances and neurotransmitters, and the prevention of peripheral and central sensitization. Some neurological disorders requiring rehabilitative intervention can show neuropathic pain resistant to common analgesic treatment. This paper addresses the effect of BTX-A in treating NP that complicates frequent disorders of the central and peripheral nervous system such as spinal cord injury, post-stroke shoulder pain, and painful diabetic neuropathy, which are commonly managed in a rehabilitation setting. Furthermore, BTX-A has an effect in relief pain that may characterize less common neurological disorders including post-traumatic neuralgia, phantom limb, and complex regional pain syndrome with focal dystonia. The use of BTX-A could represent a novel therapeutic strategy in caring for neuropathic pain whenever common pharmacological tools have been ineffective. However, large and well-designed clinical trials are needed to recommend BTX-A use in the relief of neuropathic pain.

## 1. Introduction

Pain is a diffuse, common experience in the general population involving a huge number of pathological disturbances and conditions. It is a natural protective mechanism and according to the International Association for the Study of Pain (IASP) is defined as an unpleasant sensory and emotional experience associated with actual or potential tissue damage or described in terms of such damage [1]. With regard to the underlying pathophysiological mechanisms and clinical symptoms, two main types of pain can be described, namely nociceptive and neuropathic. The former is triggered by tissue damage, whereas neuropathic pain results from a dysfunction and derangement in transmission and signal processing along the nervous system that does not depend on the continued presence of tissue-damaging stimulus and is recognized as a disease in itself [2]. Neuropathic pain (NP) is caused by structural lesion leading to functional abnormalities in the central and peripheral nervous system. It is a frequent disorder, and its prevalence is estimate to be between 6.9% and 10% in the general population [3]. NP is the main symptom of some neurological diseases such as post-herpetic neuralgia and trigeminal neuralgia and these painful conditions are, generally, included under the term. In a rehabilitation setting, different musculoskeletal disorders can show neuropathic or mixed pain, and it can be the most bothersome feature of several diseases of the nervous system that physiatrists and the rehabilitative team face in neuro-rehabilitation. This condition can complicate the recovery of subjects suffering from stroke, multiple sclerosis, spinal cord lesions, and several neuropathies promoting persistent disability and poor quality of life. The management of NP is a therapeutic challenge for clinicians and several non-pharmacological and pharmacological interventions have been proposed with variable benefit [4]. Pharmacological agents include anticonvulsants, non-steroidal anti-inflammatory drugs, antidepressants, and opioids, even if limitations can result from their side effects [5]. Fewer than 60% of patients obtain even partial relief from newer agents that have received regulatory approval for the treatment of NP [6]. As a result, the search continues for newer treatments and additional novel therapeutic targets for agents that are commonly administered for different therapeutic indications. This is the case for botulinum toxin (BTX), the use and application of which is progressively growing and extending with time. Although seven different BTX types from A through G have been described originating from “*Clostridium botulinum*”, only BTX type A (BTX-A) type B are on the market and used in clinical practice. Initially, BTX use was limited to some neurological disturbances, yet nowadays its use has expanded to a lot of medical conditions that encompass neurological, urological, gastroenterological, surgical, dermatological as well as cosmetic applications. In neuro-rehabilitation, BTX as an adjunct to other interventions can be a useful therapeutic tool for the treatment of neurologically disabled individuals. In this setting, BTX and in particular BTX-A, is predominantly used to reduce spasticity [7]. However, there is a large body of evidence describing the entry of BTXs into all types of neurons, not only motor-neurons, and a number of studies have investigated the possibility that BTX-A could be effective in treating disorders of neurological diseases not associated with muscle hyperactivity [7,8,9,10]. Furthermore, BTX-A has been used in several painful conditions including disorders characterized by neuropathic pain [11,12,13,14]. Despite both BTX-A and BTX-B being commercially available, only BTX-A has been investigated in clinical studies to relieve pain. In a neuro-rehabilitation setting, multi-faced approaches have been carried out in order to recover functional limitations that involve the nervous system. Several neurological disturbances requiring rehabilitative intervention can also show neuropathic pain which represents a further challenging obstacle in the recovery of subjects who suffer from complex sensory and motor impairments. This paper will discuss the effect of BTX-A in the treatment of NP complicated, frequent or less common disorders of the central nervous system (CNS) and peripheral nervous system (PNS) in a neuro-rehabilitation setting.

## 2. BTX-A and Action

Botulinum toxins are potent poisons present in nature produced by the anaerobic bacterium called “*Clostridium botulinum*”. Seven types of toxins have been recognized from clostridium, defined A through G. Recently, a novel toxin serotype was discovered and designated “H” [15], although its identity is controversial. The active BTX molecule consists of two chains weighing ~150,000 Daltons, in which a heavy chain is linked by a disulfide bond to a light chain [16]. Each chain has specific action; the former is responsible for neuron internalization, and the light chain binds to a specific target protein involved in the docking and fusion of acetylcholine-containing vesicles collectively referred to as the SNARE complex*,* which is responsible for vesicle acetylcholine release. BTX-A cleaves a protein of the SNARE complex termed SNAP-25 [17], blocking acetylcholine release. The derangement of this process at neuro-muscular junctions causes clinical effects consisting of muscle weakness and paralysis. To date, four formulations of BTX-A are on the market and used in clinical practice: onabotulinumtoxinA (Botox, Allergan, Inc., Irvine, CA, USA), abobotulinumtoxinA (Dysport, Ipsen Ltd., Berkshire, UK), incobotulinumtoxinA (Xeomin, Merz, Frankfurt, Germany), and a Chinese toxin Prosigne (Lanzhou Institute, Lanzhou, China). The preparations differ in the process of production, the formulations and the potencies which are determined by different biological assays based on their clinical use. BTX-B classified as rimabotulinumtoxinB, is commercially available and marketed by Solstice Neuroscience (Malvern, PA, USA) as MyoBloc in the United States and NeuroBloc (Elan Pharmaceuticals, San Diego, CA, USA) in Europe. It is important to note that the potency of a single unit is variable among the commercial formulations. The potency of 1 U of onabotulinumtoxinA (Botox) is about equal to 1 U of incobotulinumtoxinA (Xeomin), 3 U of abobotulinumtoxinA (Dysport) and 40 to 50 U of rimabotulinumtoxinB (Neurobloc). However, it is very important to recognize that this ratio of equivalence cannot be employed [18,19]. For injections, botulinum toxins type A are diluted with 0.9% sodium chloride solution. The devised volumes are variable and depend on the planned dose that the clinician intends to inject.

## 3. Neuropathic Pain: Clinical Symptoms and Mechanisms

The IASP established that NP is caused by a primary damage or dysfunction in the nervous system [20]. Neuropathic pain may occur spontaneously, either ongoing or intermittent, or may be stimulus-evoked; generally, subjects describe it as burning, itching, lancing, and numbness. However, two bothersome and prominent symptoms characterize this pain: hyperalgesia and allodynia. The former describes a condition in which a stimulus that normally causes pain produces increased pain, in other words the response is amplified, whereas allodynia concerns a condition in which a stimulus that normally does not cause pain elicits pain (*i.e.*, the pain threshold is lowered). Typically, in NP negative and positive symptoms are present. Negative symptoms are characterized by sensory reduction with a distribution that suggests a lesion or disease to a specific peripheral or central nervous system site. Positive signs include hyperalgesia, allodynia, and other sensations such as paresthaesia (an abnormal sensation that is not painful or unpleasant) and dysesthaesia (an unpleasant abnormal sensation that may be present spontaneously or evoked by a different stimulus). Diagnosis is based on the clinical disturbances and symptoms, but at present, no single diagnostic procedure allows a definite diagnosis of this form of pain. Some validated screening tools can be administered that enable a valuable identification of NP. Among these, the Visual Analogue Scale (VAS), the Numerical Rating Scale (NRS), the Verbal Rating Scale (VRS) and the Face Pain scale (FPS) are the most common instruments with which to assess pain intensity. Other less frequent measures used in clinical practice are the NP4 questionnaire, and McGill pain questionnaire short form.

The pathophysiological mechanisms that underlie NP are not fully understood. Several hypotheses have been suggested including (1) sensitization of nociceptors; (2) abnormal ectopic excitability of affected neurons; (3) disinhibition of nociception control at the spinal level network; (4) sympathetically maintained pain, and (5) CNS reorganization processes [21]. Neuropathic pain sensitization of nociceptors plays an important role. Specific stimulus with different modalities such as noxious mechanical, thermal, and chemical stimuli can activate nociceptors. They are located at the free nerve endings of unmyelinated C fibers and lightly myelinated Aδ fibers [22]. Their activation is triggered either by exogenous or endogenous substances comprising inflammatory mediators (bradykinin, prostaglandins, and other derivatives of arachidonic acid), growth factors such as nerve growth factors and neurotransmitters including neurokinins, histamine, serotonin, noradrenalin, and excitatory substances [22]. These substances are released after nerve damage and Wallerian degeneration producing characteristic neuropathic pain. Abnormal ectopic excitability of affected neurons is responsible for lancing and burning pain. After nerve injury, altered excitability of small or unmyelinated Aδ and C fibers occurs by increased concentration and instability of all types of voltage-gated sodium channels [23]. Pro-nociceptive activation and decreased inhibitory influences can increase synaptic transmission at the spinal level network. The mechanism of pain sympathetically maintained could be due to the interaction between the anatomically distinct autonomic and somatosensory systems. This condition probably includes the expression of α-adrenoceptors on primary afferent sensory fibers, which become responsive to catecholamines. Histological studies in rats have shown that after sciatic nerve injury, increased coupling of sympathetic fibers to dorsal root ganglia can occur. Sympathetic sprouting forms basket-like skeins around the somata of some primary sensory neurons and ultrastructural observations have revealed that these sprouts grow on the surface of glial lamellae that form on the neurons [24]. In humans as well, efferent sympathetic signaling seems to be associated to nociceptive input in neuropathic pain. Further mechanisms are based on increase of vasomotor activity that is mediated by the sympathetic system. This condition leads to deranged tissue microcirculation and oxygenation. Kurvers *et al.*, demonstrated a reduction of nutritive skin blood flow and oxygenation of superficial skin layers which may reflect increased sensitivity of skin microvessels to circulating catecholamine [25]. Furthermore, the acidotic milieu operates as a potent nociceptive stimulus [21,26,27]. Finally, central processing of somatosensory inputs is altered by cortical reorganization phenomena [21]. Injection of BTX-A into rat jaw muscles decreases the discharge of muscle spindles, a major sensory input which can enhance central sensitization in chronic pain [28].

## 4. BTX-A and Pain

Initially, it was observed that the effect of BTX-A on pain relief was due to the reduction of muscular hyperactivity by preventing painful ischaemia that can occur in persistent or repeated dystonic contracture such as in cervical dystonia and spasmodic torticollis [29,30,31]. However, other studies have demonstrated that the analgesic effect of BTX-A was independent from the reduction of muscular disorders and also occurred at BTX-A dosages lower than those needed for motor improvement [32], whilst also persisting after the neuromuscular benefit. On this basis, several studies were carried out that investigated the BTX-A action on nociceptive pain, particularly that following musculoskeletal disorders including arthritis [33], painful total knee arthroplasty [34], epicondilitis [35,36], plantar fasciitis [37,38], myofascial pain [39] and piriformis syndrome [40]. According to the guidelines of the Therapeutics and Assessment Subcommittee of the American Academy of Neurology [41], only epicondilitis showed significant pain relief by BTX-A injection, whereas in most musculoskeletal disorders the efficacy was uncertain [42] or neurotoxin treatment showed level B evidence. This evidence level is based on at least one class I (randomized, controlled clinical trial with objective outcome assessment) or two class II studies (randomized controlled trials as class I, but lacking some criteria of this class or prospective matched cohort study with objective outcome assessment) [43].

With regard to the underlying pathophysiological mechanisms that operate in reducing pain, although several hypotheses have been suggested, the antinociceptive effect of BTX-A remain unclear. Recently, Paterson *et al.*, hypothesized that BTX-A could block nociceptor transduction, since they observed that the intradermal administration of BTX-A in healthy volunteers produced a marked decrease in sensitivity of mechanical pain [44]. BTX-A could reduce neurogenic inflammation [45] and inhibit pain by preventing peripheral sensitization. According to a model of subcutaneous formalin-induced pain in rat, BTX-A could decrease inflammation pain (II phase) by inhibiting the release of several neurotransmitters and neuropeptides including glutamate, substance P, and calcitonin gene-related peptide (CGRP) [46] and could reduce cfos gene expression [47,48]. Repeated stimulation, inflammation or nerve injury might sensitize peripheral nerve endings that can produce excess stimulation of CNS leading to central sensitization [48]. BTX-A can inhibit primary sensory fibers promoting a reduction of peripheral sensitization, and an indirect reduction in central sensitization. A further mechanism could lie in the BTX-A axonal transport from PNS to the CNS, as demonstrated by animal experimental studies [49,50]. Other researches support that the BoNT/A anti-nociceptive effect is centrally mediated. However, until now, the mechanism of central anti-nociceptive action has remained unknown. Recently, it has been suggested that the anti-nociceptive effect of BTX-A might be associated with activity of the endogenous opioid system involving m-opioid receptors [51,52].

## 5. BTX-A and CNS Diseases with Neuropathic Pain

In rehabilitation, BTX-A is predominantly used to treat focal spasticity following CNS disorders such as stroke, brain injury, cerebral palsy, multiple sclerosis, and spinal cord injury (SCI) [7]. A number of papers have demonstrated the efficacy of BTX-A in reducing spasticity and, recently, recommendations have helped to guide and support physicians in choosing dosages and which muscles to inject [53]. Although spasticity is the most frequent motor disorder in patients requiring rehabilitation, a lot of disabling impairments and conditions can occur besides spasticity, which have scarcely any available therapeutic interventions. Additionally, neuropathic pain can complicate several CNS diseases that are resistant to the common analgesic agents. Pain is a frequent complaint in chronic post-stroke subjects, since they can develop several painful conditions such as headache, pain associated with spasticity, central post-stroke pain (CPSP), and shoulder pain. Treatment of CPSP is a challenging clinical feature and may include antidepressants, anticonvulsants, anti-arrihythmics, analgesics, and non-medication treatment, but these therapeutic strategies are often limited. Subjects with SCI and multiple sclerosis can develop painful neuropathic conditions that are managed by several therapeutic non-pharmacological and pharmacological strategies with variable benefits. However, neuropathic pain that complicates clinical features in subjects with motor and sensory impairments could be different from pure NP such as trigeminal neuralgia, post-herpetic neuralgia, and hemicranias. Since BTX-A has both sensory and motor effects, it could represent a useful therapeutic tools in neuro-rehabilitation, by reducing muscular hyperactivity and by modulating sensory derangement that can originate from damaged nervous structures. BTX-A effect for the treatment of NP complicated, frequent or less common disorders of the central nervous (CNS) and peripheral nervous system (PNS) in a neuro-rehabilitation setting will be addressed (Table 1).

**Table 1:** Botulinum toxin (BTX-A) for the treatment of neuropathic pain in neuro-rehabilitation.

**CNS diseases with associated NP**
*Post-stroke shoulder pain (PSSP)*
*Spinal cord injury (SCI)*
**Peripheral nervous system disorders and NP**
*Painful diabetic neuropathy*
*Post-traumatic neuralgia*
**Unusual painful conditions**
*phantom limb*
*stump pain*
*CRPS with focal dystonia*

Legend: CNS = central nervous system; NP = neuropathic pain; CRPS = complex regional pain syndrome.

### 5.1. Post-Stroke Shoulder Pain

Among the mentioned post-stroke painful conditions, BTX-A has only been used in post-stroke shoulder pain (PSSP). About 40% of stroke patients show PSSP [54]. This clinical complication can compromise functional gain, and contribute to longer term disability [55]. Whether the pain has nociceptive or neuropathic origin in PSSP is unclear and controversial [4,56]. Some studies have focused on the associations of PSSP with peripheral pathology, in particular tendinopathies and capsulitis attributing a nociceptive origin to shoulder pain. On the other hand, the frequent presence of damage to the spino-thalamo-cortical system and, the presence of chronic pain in the affected side could be consistent with a central neuropathic component of this type of pain. Central sensitization could occur in PSSP following contribution from variable peripheral pathologies, such as capsulitis, which are common in older people [56]. Several studies have been reported on the effect of BTX-A in relief of PSSP. Generally, BTX-A is administrated in order to obtain a reduction of spasticity as well as providing pain relief. Although the effect of BTX-A on spasticity improvement has been widely demonstrated, the effectiveness of BTX-A in reducing shoulder pain after stroke is controversial. So far, six randomized controlled trials (RCT), double-blind studies concerning BTX-A use in PSSP have been reported [57,58,59,60,61,62]. In all studies, BTX-A was injected into the spastic shoulder muscles. Furthermore, one pilot study has been published in which BTX-A was injected into the shoulder joint [63] (Table 2). The studies widely varied in size, BTX-A formulation, doses and injected muscles, associated rehabilitative interventions and functional assessment measures, making a comparison not possible. The sample size of RCTs ranged from 20 to 31 patients. In all studies BTX-A was compared to placebo, but in one it was compared to triamcinolone. Of the RCTs, three studies reported significant pain reduction after BTX-A injection [58,59,60]. Different muscles and BTX-A dosage were injected: in one [60] and two studies [58,59], 100 U (Botox) and 500 U (Dysport) of BTX-A, respectively, were used. Of those in which BTX-A treatment did not give benefit compared to controls [57,61,62], a dosage from 100 U to 200 U of BTX-A (Botox) was injected. In two studies, 100 U of BTX-A were injected into the subscapularis muscle [57,61], whereas in a remaining study a dosage from 140 to 200 U of BTX-A was injected into teres major (40–60 U) and pectoralis muscles (100–150 U) [62]. In all studies, pain was assessed by VAS (Visual Analogue Scale), except for one study in which the NRS (Numeric Rating Scale) was administered to the patients [60]. The VAS is a diffusely used measurement of pain intensity. The scale is characterized by a horizontal (HVAS) or vertical (VVAS) line that is 10 cm long. The extremes are comprised of two verbal description: 0 = “no pain”, and 100 = “worst imaginable pain”. The NRS is a segmented numeric version of the VAS in which a respondent selects a whole number (0–10 integers) that best reflects the intensity of their pain. In this tool of pain assessment, a subject selects the number (0–10) that best reflects the intensity of pain with 0 representing “no pain” and 10 “worst imaginable pain”. Pain reduction was also associated with the improvement in joint motion, particularly for external rotation of the shoulder. On the other hand, no improvement in active and passive shoulder movement was observed in studies in which no reduction of pain occurred by BTX-A injection. Rehabilitative interventions associated to BTX-A or placebo treatment were variable. In only one study, subjects underwent standard physical therapy after treatment [60]; whereas in two studies, rehabilitation was not specified [57,61]. Of the remaining studies, different rehabilitative strategies were used: non standardized physical therapy for stretching and spasticity inhibition [59], occupational therapy [62] and TENS for six weeks [58]. Follow-up was variable, ranging from 4–12 weeks. In all studies, no adverse event was observed. In the study in which BTX-A was injected into the shoulder joint, five subjects were treated with different dosages and BTX-A formulations: 100 U of Botox (2 patients), 100 U of Xeomin (2 patients) and 500 U of Dysport (one subject) [63]. Significant pain reduction to VAS: 8.7 ± 1; 1.5 ± 1.1; and 1.5 ± 1.2 at baseline, 2 and 8 weeks, respectively (*p* < 0.001) was observed. A previous systematic review affirmed that BTX-A decreased pain and improved shoulder function in patients suffering from chronic shoulder pain following spastic hemiplegia or arthritis [64]. Pain reduction was significantly greater in the botulinum toxin group compared to placebo (*p* = 0.02) at 12–24 weeks. However, large-scale multicentre RCT are needed to confirm the effectiveness of neurotoxin in this painful condition, since the mentioned studies had small sample sizes, different study designs, and mediocre quality. Furthermore, when using BTX-A in pain relief associated to spasticity, some questions arise that are unsolved; (1) injected dosage—in this respect, it is important to investigate whether lower BTX-A doses than those normally used in treating spasticity are sufficient to obtain pain reduction; (2) duration of action—it is unknown whether the effect of BTX-A on pain relief goes beyond the duration of action observed in spasticity treatment; and (3) inoculation techniques—since, intra-articular and intramuscular injection were used, it is important to know whether both modalities are efficacious or if one is more efficacious than other one.

### 5.2. Pain in Spinal Cord Lesion

Subjects suffering from SCI may experience several types of chronic pain that the International Spinal Cord Injury Pain (ISCIP) has classified in three tiers. The first tier divided pain according to pain type including nociceptive, neuropathic, other pain (*i.e.*, fibromyalgia), and unknown pain [65]. Nociceptive pain is probably the most common pain after SCI and is caused by musculoskeletal disorders. Neuropathic pain is due to a lesion or disease affecting the spinal cord as well as peripheral neuropathic pain due to a lesion of the nerve roots, including cauda equine. In the case of NP, the main characteristic is its location and generally the IASP use the terms at-level and below-level neuropathic pain [66]. Pain in SCI interferes with the patient’s participation in rehabilitation and leads to other complications, such as cognitive dysfunction, and overall poor quality of life [67]. Only case series concerning BTX-A use in treating the pain of subjects suffering from SCI, have been published (Table 3). The first report about the effectiveness of BTX-A in reducing pain due to spinal cord lesion was by Jabbari *et al.* The study described two subjects suffering from excruciating burning pain at the cervical level which interfered with sleep and activities of daily living. Subjects were treated with 100 and 80 U of BTX-A, respectively. Neurotoxin was administrated subcutaneously at multiple points (16 to 20 sites) in doses of 5 U for the site over the area of burning pain and allodynia [68]. Both subjects obtained significant improvement on the VAS lasting at least three months. Repeated BTX-A injection produced a benefit over a period of 2–3 years. Recently, Han *et al.*, described a subject with post-traumatic SCI and neuropathic pain complaining of a burning, sharp, tingling pain localized in the lower limbs, predominantly above the ankles. He was treated with 200 U of BTX-A (Botox) that was injected subcutaneously into 10 of the most painful sites of each sole at doses of 10 U per site. Significant pain relief was observed at four and 8 weeks after injection [69]. Our group described a young subject with post-traumatic SCI and a buttock ulcer that was difficult to care for due to severe painful spasms of the gluteus maximus that hampered ulcer healing. The middle component of the left gluteus maximus and the muscular zone around the ulcer were treated with 150 U and 60 U at each point of infiltration, respectively, for a total of 660 U BTX-A (Dysport). The use of BTX-A allowed for better care of the pressure ulcer, which had healed six months after the initial infiltration. Additionally, the subject claimed relief of the painful muscular spasm. In this case, even though BTX-A was mainly used to promote care of the ulcer, it broke the vicious circle of spasticity and afferent painful stimuli from the ulcer failing to heal, partially due to spasticity-induced contractures [70].

## 6. BTX-A and Pain in Disorders of the Peripheral Nervous System

Diseases and disturbances of the PNS requiring rehabilitation include a wide spectrum of motor and sensory neuropathies due to infective, vascular, immunogenic, traumatic, metabolic, toxic, iatrogenic, and hereditary origin. Among these, the most common neuropathies showing NP are trigeminal neuralgia and post-herpetic neuralgia. Painful neuropathies that can require rehabilitative intervention and treated by BTX-A, in particular painful diabetic neuropathy (PDN) and post-traumatic neuropathy, will be highlighted.

### 6.1. Painful Diabetic Neuropathy

The number of people with diabetes worldwide, currently around 374 million [71], is set to double within the next 20 years, and the increase will be most notable in the developing world. A wide spectrum of neuropathies can complicate diabetes mellitus. These disorders include mild symptomatic distal sensory neuropathy as well as severe disabling radiculoplexus neuropathy. Among these, sensorimotor polyneuropathy is the most frequent complication in diabetic subjects occurring in 10%–54% of patients with type 1 and 2 diabetes [72]. In the general population, the prevalence is widely estimated, varying from 9.6% to 78% [73,74,75,76]. A third of patients with diabetic sensorimotor polyneuropathy develop PDN, and this disorder is more prevalent in type 2 than in type 1 diabetes [77]. Diabetic neuropathy (DN) can promote disability by several causes including balance disorders [78], morphologic foot change [79], foot sore occurrence, lower extremity dysfunction [80], and gait disorders [81]. Furthermore DN and PDN are among the strongest determinants of reduced health-related quality of life in patients with type 2 diabetes mellitus [82,83]. Several rehabilitative interventions are commonly carried out in subjects with DN in order to prevent foot complications and disabling gait disturbances [84,85,86]. In these patients, neuropathic pain is a challenging condition, since it impedes rehabilitative strategies and delays functional recovery. Many drugs including anti-depressants, carbamazepine, gabapentin, and opioids have been used. More recently, the use of topical agents such as lidocaine patches and high-dose capsaicin have been introduced [87,88,89]. However, the mentioned agents might be poorly effective or not appropriate due to the lack of long-lasting pain relief and side effects [90,91]. Therefore, BTX-A has been proposed to treat the pain of subjects suffering from PDN. Animal studies have shown that in diabetic rats with allodynia, unilateral subcutaneous injection of BTX-A in the painful region of one affected limb improves allodynia in both limbs. This response can reflect a central analgesic effect of the neurotoxin [92]. Two randomized controlled (RC), double-blind trials, were reported concerning BTX-A and PDN [93,94] (Table 4). The study by Yuan *et al.*, was the first RC, crossover study investigating BTX-A in subjects with PDN. Eighteen subjects received intradermal 50 U of BTX-A (Botox) that were distributed across the dorsum of the foot, according to a grid pattern that covered a total of 12 (3 × 4) sites at dosage of approximately 4 U for injection point. Significant reduction in VAS score by 0.83 ± 1.11 at 1 week, 2.22 ± 2.24 at 4 weeks, 2.33 ± 2.56 at 8 weeks, and 2.53 ± 2.48 at 12 weeks (*p* < 0.05) after injection in the BTX-A group, as compared to the respective findings for a placebo group of 0.39 ± 1.18, −0.11 ± 2.04, 0.42 ± 1.62, and 0.53 ± 1.57 was observed, at the same time points. Recently, Gashemi *et al.*, reported a RC, double blind study in which 40 patients with diabetes mellitus type 2 were enrolled. Pain was assessed by the DN4 questionnaire, neuropathic pain scale (NPS) and VAS. The BTX-A group received intradermal 100 U of BTX-A (Dysport) injections across dorsum of the foot in 12 sites (8–10 U for site) according to a grid distribution pattern. Significant NPS scores for all items (*p* < 0.001) except cold sensation, and VAS scores (*p* = 0.01) reduction were observed in BTX-A group compared to placebo, at three weeks. No side effects occurred in BTX-A treated patients [94]. Interestingly, BTX-A was subcutaneously injected in multiple sites and a lower dosage was used. In this respect, it is important to investigate whether specific modality BTX-A delivery produces better benefits in pain relief. So far, no study comparing different administration techniques in the relief of neuropathic pain has been reported.

Despite both studies demonstrating the efficacy of BTX-A in pain relief, larger trials with longer periods of observation are needed to confirm the findings. However, BTX-A may represent a novel interesting approach treatment of subjects with PDN in neuro-rehabilitation.

### 6.2. Post-Traumatic Neuralgia

Neuropathic pain may complicate post-surgical and post traumatic nerve lesions. Two trials have been published about BTX-A use in post-traumatic neuralgia (PTN) [95,96]. Of these, only the study by Ranoux *et al.*, was considered [95] for the purpose of the present paper. This study was the first trial on the effect of BTX-A in painful post-traumatic neuropathy and consisted of a RC, double blind trial. Twenty-five subjects with neuropathic painful nerve trauma received intradermal variable doses from 60 to 190 U of BTX-A (Botox) that were injected over the painful skin area at 5 U in 20 ± 8.3 sites. Follow-up was performed at 4, 12 and 24 weeks after injections. Significant pain improvement was observed at several time points. The effect started at week 2 (*p* < 0.025) and increased for up to four weeks (*p* < 0.036) and remained stable for up to 14 weeks. In particular, a reduction of the intensity and area of mechanical allodynia, and a cold pain thresholds reduction on the painful side were observed. On the other hand, perception thresholds were not modified. Significant improvement in average pain intensity assessed at each follow-up visit (*p* < 0.0073) was also observed after BTX-A treatment. The number needed to treat 50% pain relief with BTX-A was 3.70 (2.04–23.2) at four weeks and 3.03 (1.64–21.6) at 12 weeks. Additionally, BTX-A improved general activity, mood and anxiety scores. Subjects complained of only moderate-to-severe transient pain in the site of inoculation. A long-lasting pain relief of about 24 weeks in the BTX-A treated group was also observed.

## 7. BTX Use in Unusual Rehabilitative Clinical Conditions

Less common conditions in the rehabilitation setting that could present neuropathic pain are phantom limb [97], stump pain [98] and complex regional pain syndrome (CRPS) complicated by neuropathic painful dystonia [99].

### 7.1. Phantom Limb Pain

Pathophysiological mechanisms underlying phantom limb pain (PLP) include ectopic activity at the neuroma, which drives central plasticity and sensitization [100,101]. Nociceptive stimulation from amputation and ectopic foci from active neuroma may cause an increase of synaptic firing at spinal level. This abnormal activity produces a derangement of afferent pain signals to the brain that can lead to a reduction of descendent inhibitor activity. In addition, cortical reorganization can be observed, which produces deranged circuitry and firing pattern encoding pain signals [102]. Several therapeutic strategies have been proposed with limited success [103] and different levels of evidence [104]. Case series and one RCT double-blind study have been published supporting the use of BTX-A in these painful conditions (Table 5). Initially, Kern *et al.*, reported four patients with PLP that were treated by a global dosage of 100 U of BTX-A (Botox) at 20 U for sites in the stump. In all subjects PLP relief was observed. The same group reported that neurotoxin treatment facilitated application and prosthesis use [97,98]. Jin *et al.*, reported three subjects with PLP who received BTX-A (Dysport) at doses of 300 U, 500 U, and 200 U, respectively by EMG guidance into points of strong fasciculation. In all three cases, pain intensity and pain medication decreased significantly. No side effect was reported and the duration of response lasted for up to 11 weeks [105]. The only randomized, double-blind pilot study included 14 amputees with intractable PLP [106]. BTX-A injection was compared to lidocaine/depomedrol in amputees with PLP and residual limb pain (RLP). RLP was defined as a painful condition that could occur very quickly after amputation and at a later stage due to scar and neuroma formation. BTX-A group received from 250 to 300 U of Botox, whereas the control group was treated with 1% lidocaine (AstraZeneca LP) and 40 mg/mL of DepoMedrol (methylprednisolone acetate Injectable Suspension, Pharmacia & Upjohn Co.). Both groups were injected into the muscles, subcutaneous tissues, and neuroma. The subjects were evaluated at baseline and every month after the injection for six months. No improvement of PLP was observed after BTX-A or lidocaine/depomedrol injection. However, Botox and lidocaine/depomedrol injections resulted in immediate improvements of RLP: *p* = 0.002 and *p* = 0.06 for BTX-A and lidocaine/depomedrol, respectively; and pain tolerance: *p* = 0.01 and *p* = 0.07 for BTX-A and lidocaine/depomedrol, respectively. The treatment effect lasted for six months in both groups and no side event was observed.

**Table 2:** Botulinum toxin and post-stroke shoulder pain.

Study	Design	Pts	BTX-type and doses/PT	Follow-up	Pain measures	Other measures	Adverse event	Drop-out	Outcome	Stat/S
Kong *et al.* [57] 2007	RCT, DB	22	8 pts: BTX-A (Botox) 100 U in 2 sites (50 U) of the subscapularis muscle; 9 pts placebo; not specified physical therapy	6, 12 weeks	VAS	AS; electronic goniometry for shoulder external rotation; functionality by Brunnstrom’s six stages of recovery	pain in site of injection	1 sub. of BTX-A group	No significant changes in pain or external rotation as a result of administration of BTX-A	no
Marco *et al.* [58] 2007	RCT, DB	31	14 pts: 500 U of BTX-A (Dysport) in 4 sites of the pectoralis major muscle by EMG guidance; 15 pts placebo; both groups received TENS for 6 weeks	1, 4, 12, 24 weeks	VAS	MAS; flexion, abduction and external rotation of shoulder	no adverse events in BTX-A group; 2 pts in placebo group reported transient fatigue and a moderate strength reduction in the upper extremity	2	BTX-A group showed a greater pain reduction than placebo group to VAS. In BTX-A, the mean reduction was 46.2 (SD 34.2) mm at 24 weeks, whereas the reduction was 21.9 (SD 29.4) mm in the placebo group	yes
Yelnik *et al.* [59] 2007	RCT, DB	20	10 pts: 500 U of BTX-A (Dysport) into subscapularis muscle; non standardized physical therapy for stretching and spasticity inhibition	1, 2, and 4 weeks	VAS	MAS; passive shoulder lateral rotation and abduction	no adverse event apart pain in inoculation site in 2 pts of placebo group	0	Improvement of pain in BTX-A group from week 1; significant pain reduction at week 2 (*p* = 0.042) and at week 4 (*p* = 0.007); at this time BTX-A group showed 4 points reduction, whereas 1 point was observed in placebo group (*p* = 0.025). Significant lateral rotation improvement was observed in BTX-A group compared to placebo at week 2 (*p* = 0.05) and week 4 (*p* = 0.018)	yes
De Boer *et al.* [61] 2008	RCT, DB	22	10 pts: BTX-A (Botox) 100 U in 2 sites (50 U) of the subscapularis muscle; 11 pts placebo; not specified physical therapy	6, 12 weeks	VAS	AS; humeral external rotation by means of electrical goniometer; Brunnstrom scale	nr	nr	Both the improvement in external rotation and VAS pain score were not modified by BTX-A treatment	no
Lim *et al.* [60] 2008	RCT, DB	29	16 pts: BTX-A group 100 U Botox in the infraspinatus, pectoralis and subscapularis muscles; 13 pts placebo: intra-articular injection of triamcinolone acetonide (TA); standard course of physiotherapy	12 weeks	NRS	MAS; ROM of the shoulder for the following movements: forward flexion, abduction, external and internal rotation; arm function by Fugl-Meyer scale; physician global rating	no adverse effect	4 pts: 2 in BTX-A and 2 in placebo group	Although pain improvement was observed in both groups, it was more in BTX-A than placebo group Decrease in pain was 4.2 and 2.5, respectively (*p* = 0.051). Overall ROM also improved: 82.9° and 51.8° in BTX-A and TA-treated group (*p* = 0.059)	yes
Castiglione *et al.* [63] 2011	Pilot study	5	Intra-articular 100 U of BTX-A (2 pts Botox, 2 pts Xeomin) and 500 IU (Dysport, 1 subject)	8 weeks	VAS	nr	nr	nr	At rest significant pain reduction to VAS: 8.7 ± 1; 1.5 ± 1.1; and 1.5 ± 1.2 at baseline, 2 and 8 weeks, respectively (*p* < 0.001) occurred. Significant pain reduction during shoulder passive arm abduction was also detected	yes
Marciniak *et al.* [62] 2012	RCT, DB	21	10 pts: 140–200 U of BTX-A (Botox) into the teres (40–60 U); major, pectoralis muscles (100–150 U); 11 pts: placebo; Occupational therapy	12 weeks	VAS, daily diaries; McGill pain questionnaire-Short Form	MAS; BDI; passive ROM of the shoulder by goniometer; FIM; DAS; Fugl-Meyer scale	no side effect due to BTX-A	2 pts of placebo group	Significant pain improvement in both BTX-A and placebo group was observed at 4 weeks after injection, but pain reduction in BTX-A treatment was not greater than placebo group	no

Legend: Pts = patients; Stat/S = statistical significance; PT = physical therapy; AS = Ashworth scale; MAS = modified Ashworth scale; VAS = visual analogue scale; TENS = transcutaneous electrical nerve stimulation; NRS = numeric rating scale; FIM = functional independence measure; DAS = disability assessment scale; ROM = range of motion; BDI = Beck depression inventory; nr = not reported.

**Table 3:** Botulinum toxin type A and neuropathic pain in spinal cord injury.

Study	Design	Pts	BTX-A and doses	Follow-up	Pain measures	Other measures	Adverse event	Drop-out	Outcome	Stat/S
Jabbari *et al.* [68] 2003	Case report	2	100 U and 80 U of BTX-A (Botox) subcutaneously at multiple points (16 to 20 sites, 5 U per site (16 to 20 sites) in the region of pain and allodynia	2–3 years	Pain severity reduction by VAS	nr	no side effects and no weakness	-	Case 1: VAS decreased from 8–10 to 2–3 with an 80% decrease in the frequency of more severe episodes of spontaneous pain. Case 2: significant reduction of burning pain	n/a
Han *et al.* [69] 2014	Case report	1	200 U of BTX-A subcutaneously injected into 10 most painful sites of each sole at 10 U for site	8 weeks	VAS	nr	no side effect	-	significant improvement of neuropathic pain	n/a

Legend: Pts = patients; Stat/S = statistical significance; n/a = not applicable; nr = not reported.

**Table 4:** Botulinum toxin type A and painful diabetic neuropathy.

Study	Design	Patients	BTX-A and doses	Follow-up	Pain measures	Other measures	Adverse event	Drop-out	Outcome	Stat/S
Yuan *et al.* [93] 2009	RCT, DB, placebo crossover study	20 pts	10 pts in BTX-A: intradermal 50 U of Botox over the dorsum of foot in 12 sites at dose of 4 U for site; 10 pts placebo	24 weeks	Pain severity reduction by VAS within 12 weeks	Chinese version of Pittsburgh Sleep Quality Index; Short Form 36 QOL questionnaire	mild local skin infection	2 pts	Significant pain reduction to VAS in BTX-A group; 44.4% of BTX-A patients experienced good responsive (VAS decrease ≥3) *vs.* none in placebo group; significant improvement in sleep for BTX-A group only at week 4; no significant differences in QOL between groups by Short Form 36 QOL questionnaire	yes
Ghasemi M *et al.* [94] 2014	RCT, DB, placebo controlled	40 pts	20 pts in BTX-A: intradermal 100 U of Dysport over the dorsum of foot for 12 sites at dose of 8–10 U for point); 20 pts placebo	3 weeks	DN4 questionnaire; NPD; VAS	nerve conduction velocity examinations	no side effects	-	Intradermal injection of BTX-A reduced NPS scores for all items except cold sensation (*p* = 0.05). According to VAS, 30% and 0% of patients in intervention and placebo groups have no pain after intervention (*p* = 0.01)	yes

Legend: Stat/S = statistical significance; NPS = neuropathy pain scale; VAS = visual analogue scale, QOL = quality of life.

**Table 5:** BTX-A and phantom limb pain.

Study	Design	Patients	BTX-type and doses	Follow-up	Pain measures	Other measures	Adverse event	Drop-out	Outcome	Stat/S
Kern *et al.* [98] 2003	Case series	4	100 U of BTX-A (Botox) at 20 U for sites in the stump	3 months in 2 pts	VAS	-	nr	-	In all subjects PLP relief was observed	n/a
Jin *et al.* [105] 2009	Case series	3	300 U (4 points), 500 U (12 points) and 200 U respectively of BTX-A (Dysport) by EMG guidance	11 weeks	VAS	GCI was based on a scale graded 0 = no effect to 3 = pronounced improvement	no side effect	-	Significant reduction of pain and improvement in prosthesis tolerance and gait. Repeated BTX-A injection every 3 months successfully up 7 years (case 1)	n/a
Wu H *et al.* [106] 2012	Randomized, DB	14	7 pts in BTXA group: 250–300 U of Botox; 7 pts in control group: 1% lidocaine and 40 mg/mL of DepoMedrol; both by EMG guidance	6 months	VAS; PLP; RLP	changes of the pressure pain tolerance as measured by a pressure algometer	No adverse event	2 pts at 4 months	No improvement of PLP was observed in both groups. However, immediate improvement of RLP and pain tolerance after injections for Botox (*p* = 0.002 and *p* = 0.01, respectively) and Lidocaine/Depomedrol (*p* = 0.06 and *p* = 0.07, respectively) occurred	yes

Legend: DB = double blind; Stat/S = statistical significance; n/a = not applicable; VAS = visual analogue scale; PLP = phantom limb pain; GCI = global clinical improvement; RLP = residual limb pain (pain that develops very quickly after an amputation due to surgery, and the later stage due to scar and neuroma formation).

### 7.2. Complex Regional Pain Syndrome and Painful Dystonia

Several symptoms are associated to pain in complex regional syndrome (CPRS) including sensory autonomic, trophic, and motor abnormalities. IASP recognizes type I and II CPRS. The pathophysiological mechanisms producing this disabling condition are unknown, although inflammatory, vasomotor dysfunction, and abnormal neuroplasticity have been suggested [107]. About 25% of patients with CPRS type I can show focal tonic dystonia [98]. Controversies arise about the underlying mechanism of associated movement disorders including psychogenic [108], genetic HLA association origin [109,110], and PNS neuropathy of small-fibers characterized by dysfunction of C and Aδ-fibers [111]. Orthoses such as splints or plaster casts have been used to prevent deformity and support function but are often ineffective and may even increase involuntary movements [112]. Kharkar *et al.*, retrospectively investigated 37 subjects with CRPS. Pain scores were recorded on an 11-point Likert scale, with 0 indicating “no pain” and 10 the “worst imaginable pain”. Participants with spasm or dystonia in the upper limb girdle muscles were referred for BTX-A treatment. BTX was injected by EMG guidance into the specific upper limb girdle and neck muscles that were selected by patient complaints, hypertrophy, spasm and/or tenderness on palpation. BTX-A total dose used was 100 U in each patient. Significant pain relief was observed in almost all treated patients: mean pain score decreased 43% (8.2 ± 0.8 to 4.5 ± 1.1, *p* <0.001). One patient had transient neck drop after the injections [113]. BTX-A effect on pain relief could be due to the reduction of the sympathetically maintained pain mechanism. The authors postulated that the pain relief following intra-muscular BTX-A injection was multifactorial, including the relief of neurogenic inflammation, and relaxation of dystonic muscles, which might decrease the afferent nociceptive barrier from sensitized A-delta and C fibers.

## 8. Limitations in the Use of BTX

Although there is growing use of BTX-A in clinical practice, information to guide the choice of toxin remains limited. Currently, injected BTX-A doses widely vary and are based on several aspects including experience of the practitioner, expert opinion, as well as the formulation of BTX being used and the individual patient’s response. A drawback for BTX-A therapy is its high cost and the transient nature of the toxin. Since, BTX has a duration of effect that lasts from a few weeks to six months [14,103], it requires less frequent administration than other medications. In this respect, it has been reported that BTX-A clinical benefits outweigh the high cost of this agent, particularly in post-stroke spasticity resulting in a cost-effective therapy [114]. However, no data have been reported on this issue in treating other disturbances. Furthermore, BTX-A treatment could cause unpleasant focal and general side effects characterized predominantly by distant weakness. Interestingly, no severe adverse event was reported in treating neuropathic pain apart from mild pain in the inoculation site, even if BTX-A dosages, normally injected in treating muscle spasticity, were used in disturbances such as PSSP and PLP. One patient had transient neck drop after the BTX-A injections in treating CRPS. It is possible that experienced operators and injection modalities by EMG guidance could have reduced side effects. Lower BTX-A dosages such as those injected in PDN could also explain the lack of adverse events. Before performing BTX-A injections for therapeutic purposes, the expected risks and benefits for each patient must be carefully considered. The development of neutralizing antibodies (NAbs) can eliminate the effects of BTX-A. Antibody formation against BTX proteins is one of the reasons for therapy failure, particularly in treating spasticity and dystonia. The development of NAbs are facilitated if repeated injections and high dosages of BTX are used, independently from the treated disturbances. NAbs has been also observed in subjects who underwent BTX injections for non-motor disorders such as sialorrhea. However, no data have been reported about the formation of NAbs against BTX-A in treating neuropathic pain.

## 9. Considerations and Future Directions

A careful evaluation of functional limitation, goals, and expected outcomes should be evaluated in subjects with complex disabling neurological dysfunction, prior to initiating BTX treatment. The main objective of the rehabilitation process in disabled people is the improvement of activities, participation, and quality of life. BTX strategies should be viewed as adjunct measures to other common strategies in achieving the best functional outcome. Neuropathic pain can complicate the course of several central and peripheral neurological diseases requiring rehabilitation. This condition can prevent rehabilitative processes, favoring disability and poor quality of life. In a rehabilitation setting, as well as common pharmacological agents for the relief of NP, non-pharmacological interventions can be used, such as acupuncture, transcutaneous electrical stimulation (TENS), spinal cord stimulation, and peripheral nerve stimulation. Previous reviews have been published concerning BTX-A in treating neuropathic pain, but they focused predominantly on the single injection technique [115], mixed neuropathic painful musculoskeletal and neurological diseases [14,68,116,117] and pure neuropathic condition such as hemicranias, post-herpetic neuralgia, and trigeminal neuralgia [34]. The present review addresses neuropathic pain that complicates neurological diseases requiring rehabilitation and it can support physicians and rehabilitative staff in further novel therapeutic approaches for complex disabling conditions which could be scanty of therapeutic interventions. A recent systematic review according to Grading of Recommendations Assessment, Development, and Evaluation criteria (GRADE) [118] including the analysis of publication bias [119] and unpublished trials showed weak quality of evidence on the effectiveness of BTX-A in neuropathic pain and recommended this agent as a third-line treatment [120]. Therefore large, well-designed clinical trials are needed to clearly demonstrate BTX-A effectiveness in patients suffering from this painful condition. However, the present review showed that BTX-A could represent a novel therapeutic strategy in caring for several neuropathic painful conditions that need neuro-rehabilitation. Since several reports were case or case series studies, large and well-designed clinical trials should be planned to unequivocally demonstrate BTX-A effectiveness in these disturbances. Furthermore, some considerations and questions arise when using BTX-A in these disorders that particularly concern site and injection techniques as well as injected dosage. Since variable inoculation techniques including intramuscular, sub-cutaneous and intra-articular delivery were used, it is important to examine whether all modalities are equally efficacious and safe or whether one result is superior to the others. Likewise, variable BTX-A formulation and dosage were injected. In this respect, it would be very important to investigate whether lower dosages than those normally used in treating muscular hyperactivity were equally efficacious and whether the benefit on pain relief has different durations of action compared to that observed for the treatment of spasticity or dystonia. Therefore, well-designed, large clinical trials are needed to address these unsolved questions.
